# Linking the Urban Environment and Health: An Innovative Methodology for Measuring Individual-Level Environmental Exposures

**DOI:** 10.3390/ijerph20031953

**Published:** 2023-01-20

**Authors:** Kimon Krenz, Ashley Dhanani, Rosemary R. C. McEachan, Kuldeep Sohal, John Wright, Laura Vaughan

**Affiliations:** 1 The Bartlett School of Architecture, Faculty of the Built Environment, University College London, London WC1H 0QB, UK; 2 Bradford Institute for Health Research, Bradford Teaching Hospitals NHS Foundation Trust, Bradford BD9 6RJ, UK

**Keywords:** environmental exposure, built environment, public health, geographic information systems, accessibility

## Abstract

Environmental exposures (EE) are increasingly recognised as important determinants of health and well-being. Understanding the influences of EE on health is critical for effective policymaking, but better-quality spatial data is needed. This article outlines the theoretical and technical foundations used for the construction of individual-level environmental exposure measurements for the population of a northern English city, Bradford. The work supports ‘Connected Bradford’, an entire population database linking health, education, social care, environmental and other local government data over a period of forty years. We argue that our current understanding of environmental effects on health outcomes is limited both by methodological shortcomings in the quantification of the environment and by a lack of consistency in the measurement of built environment features. To address these shortcomings, we measure the environmental exposure for a series of different domains including air quality, greenspace and greenness, public transport, walkability, traffic, buildings and the built form, street centrality, land-use intensity, and food environments as well as indoor dwelling qualities. We utilise general practitioners’ historical patient information to identify the precise geolocation and duration of a person’s residence. We model a person’s local neighbourhood, and the probable routes to key urban functions aggregated across the city. We outline the specific geospatial procedure used to quantify the environmental exposure for each domain and use the example of exposure to fast-food outlets to illustrate the methodological challenges in the creation of city and nationwide environmental exposure databases. The proposed EE measures will enable critical research into the relationship and causal links between the built environment and health, informing planning and policy-making.

## 1. Introduction

The influential role of the built environment in shaping our health and well-being is increasingly being recognised [1]. Growing evidence suggests strong relationships between exposures to environmental characteristics such as air pollution [2,3,4], noise [5,6,7], green space [8,9,10] and greenness [11,12,13], public transport [14,15], walkability and street centrality [16,17,18], unhealthy food [19,20,21], or indoor dwelling qualities [22,23] and health outcomes across the globe. While the detrimental effects of environmental exposures have been broadly demonstrated in numerous cases, there is also growing evidence for positive effects of built environment features. Individual features of the built environment have been linked to increased physical activity [24], active travel [25], or lower levels of obesity [26]. For example, neighbourhoods that are walkable are associated with increased physical activity and other positive health outcomes [27,28]. Moreover, the built form and shape of green spaces have an impact on the number of walking trips fostering physical activity [29]. There are also occasions where the built environment features can be both beneficial and detrimental, such as the proximity to busy roads being supportive for active travel, but bad through their potentially high level of air pollution. Having a greater precision for environmental indicators would enable a more nuanced analysis of the relationship between the built environment and health than has been possible to date.

A major limitation of the existing ways of constructing large-scale spatial indicators is that they focus on aggregate levels such as administrative boundaries or postcodes, and do not take into account individual-level exposure [30]. Increasing evidence shows that exposures perceived at the street level (e.g., air pollutants) can have particularly detrimental effects on health [31,32]. Such negative effects can occur even at low levels (i.e., short-term and low concentrations) of environmental exposure [33]. Exposures modelled at national scales have been found to significantly underestimate the real-world exposure perceived at the street level [34]. Evidence from small-scale studies indicates that characteristics of the built environment, such as higher densities of buildings forming street canyons, may lead to increased levels of exposures [35]. This highlights the need for better quality spatial data at smaller scales to differentiate between conditions at the finest, street-level scale, since quantifying environmental exposures at the scale at which they are experienced by individuals (rather than in arbitrarily designated geographies) can provide crucial information for improving the knowledge on the impact of environmental exposures on health and well-being. Such knowledge will inform spatial planning decision-making, e.g., in guidance for targeted modifications of the built environment that accounts for its complexity.

Agreement over the importance of individual features had been varied in the past. A recent meta-narrative review by Ortegon-Sanchez et al. [36] on the relationships between the built environment and child health, identified vast inconsistencies in the way in which neighbourhood characteristics are measured and conceptualised within health studies. These inconsistencies might explain the existing contrasting evidence for specific environmental features and health outcomes, such as between quantifications of food environments and obesity [37], or between urbanicity and schizophrenia [38]. In the latter, advances are being made in improved precision of measuring built environment features, and it is to this literature that we hope our work will contribute [39].

Finding relationships between specific built environment features and health outcomes opens the possibility for targeted planning and policy interventions. Yet, it is imperative for such interventions that decision-makers understand the precise built environment features involved to avoid adverse negative effects of policies to improve health outcomes [40]. Modifying the built environment effectively requires an intrinsic knowledge of the importance of individual features, as well as their interrelated effect on health. However, causal links between some built environment features and health outcomes are still to be proven definitively, despite many attempts to do so [41].

There are significant differences and heterogeneity between the physical and environmental characteristics of neighbourhoods of a similar population density within cities (e.g., dispersed high-rise and dense low-rise buildings). A large part of the field of urban planning is dedicated to the quantification of such differences measuring the geographical, form and functional properties of the built environment and their relationships [42,43]. Such nuanced and detailed measures of the built environment have not yet been systematically incorporated into the research on public health. We extend the argument by Cyril et al. [44] of ‘an urgent need for health studies to standardise measures of urbanicity’, to ‘the urgent need for comprehensive and detailed standardised longitudinal measures of the built environment’ for health and public health studies. Challenges for health research in understanding the importance of environmental features not only arise through the difficulties in the effective quantification of these but by their complex intersection with additional social, cultural or economic factors.

Existing and emerging large-scale linked population datasets which track health over time provide rich individual-level information on health and socio-economic factors. Combining such datasets with high-resolution geospatial environmental information of exposures, including the length and extent of exposure, holds the potential to improve the value of existing population datasets for the discovery of causal relationships between built environment characteristics and health outcomes. Geospatially-enriched population data also enables investigations into the interaction effects between different built environment characteristics on health.

We aimed to construct a detailed set of built environment indicators for every address in Bradford, accounting for the limitations previously identified. This paper describes their development, highlights key challenges, and outlines potential pathways to accelerate discoveries. We hypothesise that by linking meaningful built environment features to longitudinal whole population databases, researchers will achieve (a) an improvement in the methods for correlating the built environment with health and social outcomes; (b) will support the production of new knowledge on how the built environment contributes to diseases; and (c) in linking to cohort data, to improve the understanding of how and when the built environment shapes long-term health outcomes that are the result of growing up in unhealthy environments.

We quantify a series of environmental exposures at an individual level across 11 different domains and link these to individual-level health data to investigate relationships at a high level of detail. Environmental information is gathered for a whole population and we outline how these can be linked to a longitudinal birth cohort in Bradford, England. Our exposure indicators capture qualities across air pollution, greenspace and greenness, public transport, walkability, traffic, buildings and the built form, street centrality, land use, and food environments as well as indoor dwelling quality domains. We demonstrate our methods on data around the year 2020 and outline how the proposed methods hold the potential to be created longitudinally for any year between 2007 and 2022, and in future years, if data are available.

Section 2 presents our theoretical approach, the methodologies of increased spatial precision and the standardised conventions in quantifying the built environment, Section 3 provides critical indicators to inform the existing contrasting pieces of evidence within health research, and Section 4 discusses challenges in creating city and nationwide datasets, and outlines potential pathways to accelerate discoveries in public health through the application of large-scale environmental exposure and linked population datasets.

## 2. Materials and Methods

Increasing evidence points to the importance of individual built environment characteristics on health and human behaviour, especially those that can be linked to encouraging physical activities [45,46]. In line with this recognition, we measured exposure through the perspective of an individual perceiving the built environment as they traversed and used it. We utilised a person’s address as the starting point to measure the likeliness of a person to interact with their immediate surroundings, and for simulated journeys through the streets, the neighbourhood and to urban features. We used this lived environment to quantify the exposures and to aggregate these at the address, which also formed a point of linkage to the individual-level health information from historical data held in a whole population database and sourced from general practitioners (i.e., GPs, namely, family doctors) in the city of Bradford.

In doing so, our approach differed from the existing large-scale environmental exposure indicator datasets that are based on arbitrary geographic boundaries [30], by capturing the granular differences of the built environment at the level they are experienced. This is an important distinction as, e.g., postcodes and administrative boundaries are generally affected by a modifiable area unit problem [47], and their centroids can introduce unobserved biases into the precision of computed proximities. For example, our analysis has shown that the location of address points within the rural postcodes in Bradford can be as much as 6 km away from the actual postcode centroid—an observation which particularly on nationwide scales will have substantial effects on the analyses. We acknowledge that defining what specifically constitutes a person’s neighbourhood can differ widely between individuals [48,49], which is why approaches that utilise catchment areas suffer from arbitrary cut-off points. To address such limitations, we used a series of different metric distance radii and proposed a set of geospatial methods that aim to simulate a person’s lived environment, such as the introduction of distance decay functions (outlined in Section 2.4).

The underlying premise for our environmental exposure indicators (EI) is that, in the lack of large-scale individual-level empirical data on movement and interaction, we can measure environmental exposure by simulating potential interactions using methods from transport and urban planning. We argue that, e.g., the usage of green space will be more likely to occur if the space is close to a person’s house, or that a person will be less likely to be exposed to air pollution if their home environment has clean air. However, we acknowledge that interaction with and exposure to the environment is not solely influenced by accessibility and proximity, but also by individually-differing behaviours, attitudes and norms [50], whether they be cultural (e.g., food consumption), social (e.g., family structure), or economic (e.g., car ownership shaping how and where a person lives, works, and socialises). Keeping this in mind, we quantified the accessibility to urban features both according to the direct proximity to them and as a cumulative opportunity for exposure to them; in other words, we modelled an individual’s potential of having an encounter with any given feature, as well as their proximity to it.

While the EIs proposed in this article were tailored to be utilised within the Connected Bradford database (see Section 2.1), the methodology has been designed to enable an application to any data sources holding individual-level address information (subject to the necessary approvals), e.g., longitudinal cohort studies including those where the participants’ address information is only available at the level of the postcode (or outside of the UK to their equivalent, e.g., zip codes). The underlying datasets have been available annually since 2007 allowing a longitudinal construction of the proposed environmental exposure indicators. We will illustrate how the EIs can be linked to other datasets, such as longitudinal cohort studies using the example of the Born in Bradford (BiB) multi-ethnic family cohort study established in 2007 [51] (see Section 4).

### 2.1. Setting

**Figure 1:**
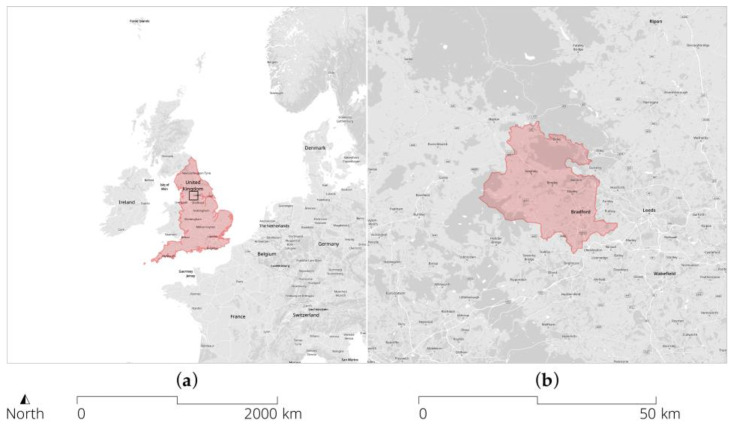
Context map showing England, located in Western Europe (**a**), and the City of Bradford Metropolitan District within the north of England (**b**). © OpenStreetMap contributors and © OS and Crown copyright 2022.

Bradford, a city of 546,400 people [52] located in West Yorkshire in the north of England (Figure 1), constitutes a particular case due to its significant number of people with limited social mobility, low educational outcomes, and poor health. The city is among the 15 most deprived districts in England, with more than a third of its administrative areas within 10% of the most deprived areas nationally [53]. These inequalities are exacerbated by low levels of employment and income. Bradford’s population is “dominated by younger age groups” [54], and with a distinctively high percentage of South Asian origin residents (90% of whom are from Pakistan) [51]. Meanwhile, the average life expectancy rate at birth for residents in the Bradford District (2018–2020) is lower for men at 77.3 years than for women at 81.5 years, and around 2 years lower than the national life expectancy, which is 79.0 and 82.9 years, respectively [55]. Set in this context is the Connected Bradford Whole System Data Linkage project [56], a whole population database that combines de-identified, longitudinal, near-to-real-time data from different organisations for approximately 800,000 citizens across the Bradford region in the north of England. The database uses pseudonymised NHS numbers (unique identifiers for every person registered with the National Health Service (NHS) in the country) and other data variables to combine a series of primary care data from general practices with community care and secondary care data, socio-economic information such as social care, education and housing, and benefits data from local authorities, as well as crime data from West Yorkshire police. This includes, e.g., variables on a patient’s medication, their clinical history, or emergency care usage. Connected Bradford allows, hence, the tracking of routine health outcomes such as a patient’s weight or body mass index (BMI), the frequency of their health and mental health service use, the prescription of specific drugs (e.g., asthma medication), or school attendance, among many others (see [56] for a comprehensive list and description).

### 2.2. Data Linkage

The data linkage between our EIs and the Connected Bradford database was achieved through a five-step process, i.e., (1) we derived environmental EIs for each residential Unique Property Reference Number (UPRN) in Bradford; (2) we received person identifiable information (i.e., historic address information) including pseudonymised NHS numbers from the Bradford Teaching Hospitals NHS Foundation Trust; (3) we georeferenced this historic address information and linked it to the UPRNs (as outlined below) and included EI data for each; (4) we pseudonymised the UPRNs and removed all the identifiable information; and (5) we provided Connected Bradford with a dataset of pseudonymised NHS numbers, pseudonymised UPRNs and linked EI data.

For step 3, we conducted large-scale georeferencing of the available patient address information from all the participating GPs within Bradford. The data comprised address information from 1950–2022 including more than 22,500,000 address rows for 1,110,000 unique pseudonymized NHS numbers. We used this address information in conjunction with Ordnance Survey (OS) AddressBase Premium data (which provides up-to-date accurate information about addresses and properties in the UK) to match a patient address record to its respective UPRN. UPRNs are unique identifiers for every addressable location in the UK. We matched the UPRNs to each patient address by adapting the open-source address-matching Oracle and R package addressMatchR. The code was rewritten to run solely in the open-source programming language R and further adaptations were made to tailor the approach to the particularities of GP address information, requiring an extended simplification and cleaning of the input data (an open-source version of the code can be obtained via the GitHub repository https://github.com/kimonkrenz/CBHE (accessed on 14 December 2022)). The result was a historical address record for every patient, linked to their respective NHS number within Connected Bradford. This enabled the linkage of environmental information to a patient and their health and other wider determinants of the health data captured in the Connected Bradford database via UPRNs. Sohal et al. [56] provide details of the granted ethical approval (IRAS ref: 239924, CAG ref: 18/CAG/0091 and REC ref: 18/YH/0200) and the implemented mechanism to prevent the intentional or unintentional re-identification of individuals within this dataset.

In parallel, we used the geographic information and address classification available within the OS AddressBase Premium dataset to identify all residential addresses, their UPRN and their location in Bradford. EIs could, thus, be generated for every residential address in Bradford and subsequently linked without the need to access patient information in the process. Our methodology also allowed the computation of EIs for alternative address information or UPRNs, such as schools, libraries or workplaces, given these are linked to a person.

### 2.3. Built Environmental Data Sources

The base for our analyses comprised high-resolution vector-based geospatial data from OS (i.e., OS MasterMap Topography, OS MasterMap Highways Roads and Path, OS AddressBase and OS Points of Interest (POI) data). The OS datasets provided the most detailed, comprehensive and up-to-date view of Great Britain’s landscape, the built environment and the land-uses. This high-resolution vector-based OS information has been available in a comparable and complete format since 2007. We used this geospatial information from 2021 to construct a street network model through which we measured the proximities to environmental exposures. This network comprises all the publicly accessible roads, and also includes all the footpaths through towns and cities to comprehensively capture how and where a person might walk. The network is dissected into 20 m long segments, of which each start and end node can constitute the beginning or end of journeys to and from urban features. The environmental exposures were based on either primary data collection, modelled information, or where appropriate, secondary data. This included vector-based information on the location of urban features (e.g., POI, National Public Transport Access Nodes (NaPTAN)), entrance/access points (e.g., OS Open Greenspace), the parts of streets, pathways and their properties (OS MasterMap Highways), 3D building information (OS MasterMap), and image-based satellite data (Landsat 8–9), as well as additional data sources capturing the indoor (e.g., energy performance certificates (EPC)) and outdoor qualities (e.g., local authority traffic and air quality data).

### 2.4. Construction of Exposure Variables

**Figure 2:**
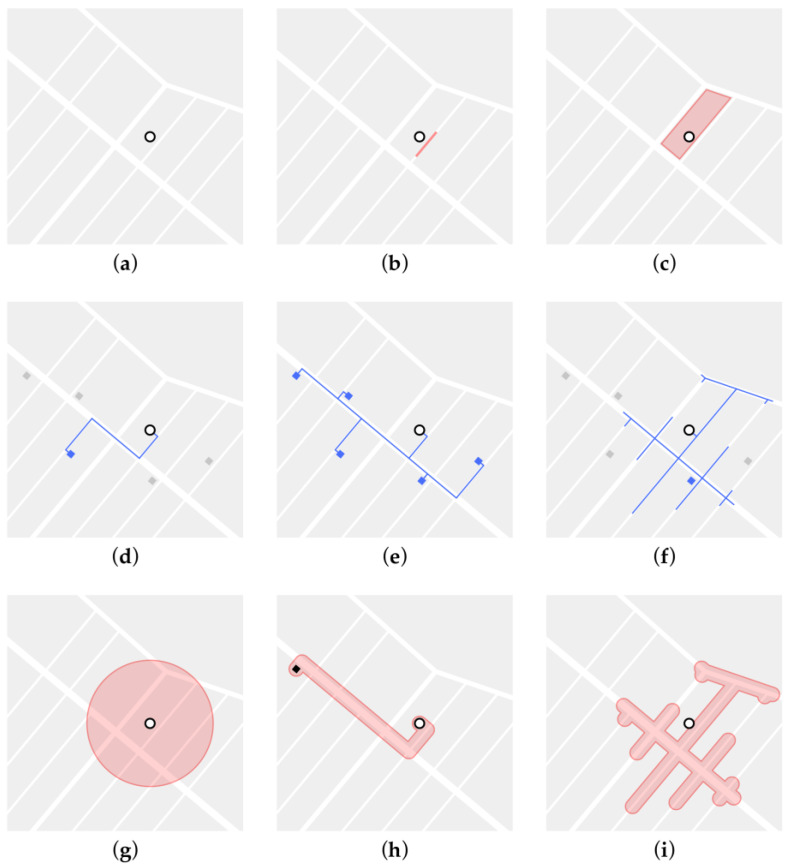
Different methods quantifying environmental exposure at varying spatial relationships: at (**a**) an address; (**b**) an address street; (**c**) an urban block containing the address; from an address to: (**d**) an environmental feature; (**e**) all environmental features; (**f**) environmental features within a catchment area; a buffer around (**g**) an address; (**h**) a route from an address to an urban feature; (**i**) a catchment area from an address.

Utilising the Geographic Information System, we derived a series of measures quantifying exposures to and within the built environment. For this, we defined nine different types of spatial relationships through and at which an individual can be exposed to the environment and features of the built environment (Figure 2). This approach took account of the most basic forms through which humans interact with the environment; both static (e.g., at the place of residence) and dynamic (e.g., while walking along a route). Therefore, these nine spatial relationships can be divided into exposure at specific locations, i.e., (a) at and within the residential home, (b) at the residential street next to the home, (c) at the urban block (i.e., the area enclosed by streets and paths) containing the residential home, (g) at a circular area surrounding the residential home, as well as exposures at places of potential interaction, i.e., (h) along a route to an urban feature or (d) to the closest urban feature from the residential home (e.g., parks entrances), (e) along a route to all, or the average distance to all the urban features of a specific type, (f) to urban features within a catchment area, and (i) to properties of routes within a catchment area. We used an open-source PostgreSQL object-relational database in combination with PostGIS, a spatial database extender, for the construction of a geospatial database and the calculation of the environmental exposure variables. PostGIS allows for fast and large-scale spatial-based queries enabling a simultaneous computation of the spatial metrics for an entire city and potentially an entire country.

**Figure 3:**
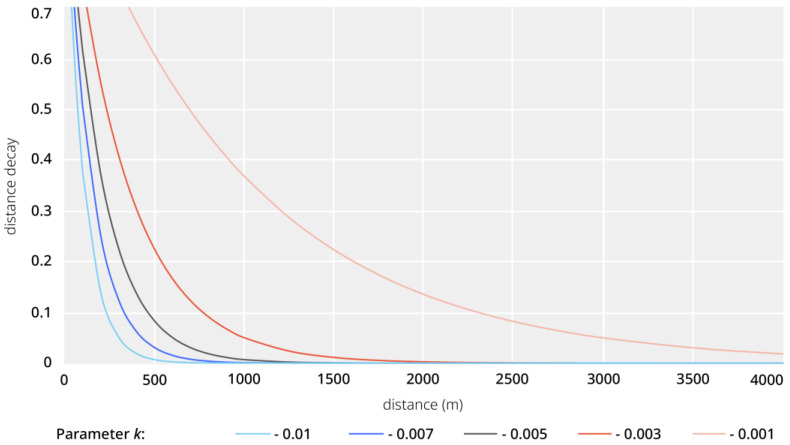
Plots of different exponential decay functions and their effect on distances in meters.

In addition to the outlined spatial relationships, we introduced four different distance types used in our analysis, i.e., (1) the metric distance as the crow flies (ignoring particularities of the spatial configuration), (2) the metric distance through the street network (representing how a human travels through and perceives the environment), (3) the angular distance through the street network (representing how a human navigates the environment), and (4) the distance decay metric distance through the street network (incorporating the effect of distance into the likelihood of an interaction between a human and the environment). Distance decay functions are standard methods in pedestrian accessibility modelling within the field of urban and transport planning. The core aim of these approaches is to incorporate the effect of distance in the analysis, i.e., the decreasing importance of an urban feature to a person with an increasing distance from it and to overcome the limitations of choosing otherwise necessary distance cut-off points. Additionally, distance decay functions provide a method to continuously decrease values until converging to zero, rather than an abrupt cut-off as in the buffer or catchment area approaches. While a plethora of different distance decay functions has been proposed, there is little statistical difference between these [57]. For the environmental indicators that use the distance decay metric distance (*D*), we have selected the following exponential distance decay function:

(1) $$D=e^{-kd},$$

where *d* is the distance in meters and *k* is a decay parameter. We applied this function to distances between addresses and urban features at varying decay parameters (Figure 3) to account for the potential differences in user groups (e.g., children, families with prams, and the elderly).

### 2.5. Selection of Environmental Domains

We selected a series of 11 environmental domains for which we generated exposure indicators. This selection was based on the existing evidence pointing to potential associations between each of these built environment domains and health outcomes, and it was informed by the policy priorities of the City of Bradford. Where possible, we sought to refine the existing measurements in order to build on the existing research in this domain and to offer a methodology that could be adopted relatively straightforwardly. The following section will outline the reasoning for each domain, their data source and the specificities for their calculation. Unless further specified, see Section 3 for details on the various spatial relationships and scales used for each domain.

#### 2.5.1. Air Quality

Air pollution has long been associated with negative health outcomes [2,3,4]. Population-wide analyses into the relationships between exposure to air pollution and health often utilise aggregate information, such as the UK Emissions data from the Department for Environment, Food and Rural Affairs (Defra) which features a 1 × 1 km resolution [58]. Villeneuve and Goldberg [59] highlight this as a common shortcoming in studies and advocate for high-resolution spatial datasets due to the variability of air pollution at small scales. We addressed this need by utilising high-resolution (1 × 1 m) air pollution data from 2018 on the annual average concentration of the particulate matter (PM) of 10 and 2.5, as well as nitrogen oxides (NOx) from the City of Bradford Metropolitan District Council. This dataset was the result of an air quality model (i.e., a Ricardo-AEA Rapid-Air complex dispersion modelling), which estimates the concentration at a 1 × 1 m resolution. The model utilises information gathered from more than 200 automatic and non-automatic monitoring sites across Bradford’s urbanised areas (see [60] for a detailed description of the underlying air quality monitoring data and the location of monitoring sites), in conjunction with local data on industrial sites, vehicular and train traffic, background concentrations and domestic heating activities. For rural areas where air pollution is not a concern and small-scale monitoring stations and tubes are scarce, we used the UK emissions data. We note that research designs that are interested in traffic-related pollution might be better placed to use traffic variables.

We calculated the average and maximum values of the PM of 10 and 2.5 and NOx for the buffer and catchment-based buffer areas at varying distances.

#### 2.5.2. Road Traffic

The effects of road traffic, such as noise [61] and air pollution (see Section 2.5.1), can have a series of adverse effects on health and quality of life which is increasingly being recognized by local authorities. Road Traffic is generally measured at the street level through annual manual traffic counts at various locations (e.g., major and minor roads) and aggregated to annual average daily flows (AADF). In the UK, streets that are not covered by manual counts are estimated in street-level estimation models. Such modelled data is often insufficient in capturing the temporary fluctuations during the day common to traffic flows and does not cover sufficient information for small local roads. To overcome this limitation, we utilized the UK-wide Trafficmaster data, which has tracked the GPS information from more than 135,000 vehicles in 1 to 10 s intervals, since 2019. The data is purchased by the Department for Transport and is available to local authorities across the UK. Besides the count data, the data also contains information on the average speeds and free-flow speeds per street segment. We used these to derive a ratio-based congestion variable as follows:

(2) $$C=\{\begin{array}{cc} 1\; & x\geq a,\\ \frac{x}{a}\; & otherwise \end{array},$$

where *a* is the free-flow speed and *x* is the average speed during the observed period.

We calculated the annual average and maximum bidirectional count of vehicles, as well as the maximum and average level of congestion for three time periods (i.e., peak morning (07:00–9:00), off-peak (10:00–16:00) and peak evening (16:00–19:00)) during weekdays. Besides measuring the count and congestion at the address street, we also aggregated the data for a 300 m catchment area around the address.

#### 2.5.3. Greenness and Greenspace

Numerous studies have highlighted associations between green space [8,9,10] or greenness [11,12,13] and health outcomes. The general method for measuring the degree of greenness is the normalised difference vegetation index (NDVI). This pixel-based metric estimates the density of green vegetation within satellite imagery. A high pixel resolution is critical to deriving meaningful small-scale estimations, and previous research [62] has demonstrated that a 30 m resolution provides sufficient detail. The NDVI can be calculated from historic and globally available satellite data, such as the USGS Landsat 8 product (2013–present), as follows:

(3) $$NDVI=\frac{NIR-RED}{NIR+RED},$$

where *NIR* is the light reflected in the near-infrared spectrum (Landsat 8 Band 5) and *RED* is the light reflected in the red range of the spectrum (Landsat 8 Band 4). We used cloud-free data from May 2020, the greenest month on record.

We calculated the average NDVI within varying radii and walking distances. While the NDVI can capture the general level of greenery in an area (sometimes referred to as availability), it lacks information on the type and accessibility of usable green spaces. For this reason, we calculated the accessibility by computing the distance from an address to the closest entrance points of green spaces. We utilized the OS Open Greenspace dataset, which includes information on entrances, area sizes and the following green space classifications: public parks or gardens, allotments or community growing spaces, cemeteries, play spaces, religious grounds, bowling greens, golf courses, other sports facilities, playing fields, and tennis courts. We followed the recommendations from the Accessible Natural Greenspace Standard for England [63] and the WHO [64], and computed the counts of green spaces within various distances, then combined these with two refined measures: a distance decay weighted count, as well as the distance weighted size. In doing so, we were able to capture the likelihood of interaction with a green space of a certain class by a person’s proximity to spaces considering that the interaction grows with size.

We calculated measures for all the green spaces and each class respectively, whereas the individual green space classes could be combined into new variables through an addition. Specifically, we counted the number of green spaces of 2 ha within 300 m, 20 ha within 2000 m, and 100 ha within 5000 m. We then counted the number of green spaces within varying radii, and we calculated the distance decay weighted counts and greenspace area for varying parameters.

#### 2.5.4. Public Transport

The use of public transport has, in various ways, been associated with better health [14,15]. Most studies interested in public transport accessibility quantify the proximity to public transport stops using an as-the-crow-flies distance, walking distance or travel time. We adapted these approaches and calculated the proximity to public transport stops using the 2022 National Public Transport Access Node (NaPTAN) database. The NaPTAN provides historic (1998–present) and nationwide information on the points of access to public transport. The spatial information ranges from a stop’s location to the entrance points of larger stations and it includes a classification for each stop/entrance (i.e., bus, coach, metro, rail, and airports).

We calculated the walking distance to public transport access points for each class, including the closest available point. We counted the number of public transport stops within varying radii, and we calculated the distance decay weighted counts for varying parameters.

#### 2.5.5. Walkability and Land-Use Intensity

Providing walkable and diverse neighbourhoods are two core policy priorities for local authorities aiming to deliver on sustainability targets. The degree to which an area is walkable has been linked to increased walking behaviour and better health [16,17,18]. A common way to evaluate the walkability of a street is to utilise street centrality measures (see Section 2.5.6.), as these can form an effective method, particularly with a lack of additional data sources. For this work, we selected a methodology that utilised additional datasets to derive better estimations. Specifically, we used a pedestrian demand model [65] which was based on datasets used in other environmental exposure variables (i.e., OS MasterMap, OS AddressBase Premium, OS Highways, and NaPTAN), of which comparable datasets can be found worldwide. The model was based on a combination of land-use intensity, transport accessibility, street network centrality and residential population density, which resulted in a raster-based geographic data surface at a resolution of 25 × 25 m generated through interpolation. We generated this model for Bradford and used the raster-based output as the base for aggregating the walkability variables from 2018. In addition, we also included the land-use intensity subcomponent as an individual variable. The land-use intensity was based on Shannon’s Diversity Index, which is calculated as follows:

(4) $$H=-\sum_{i=1}^{s}p_{i}\ln p_{i},$$

where *H* is Shannon’s Diversity Index, *i* is the proportion of one land use area of all the land uses present and *p_i_* is the total value of the land use area. We further use *H* to account for the equitability of the mix. For a detailed description see Dhanani et al. [65].

We calculated the average and maximum walkability and land-use intensity for varying radii.

#### 2.5.6. Street Centrality

Street centrality, or street network centrality, is an established metric to quantify the spatial configuration and urban morphology of an area by computing a relative centrality metric for every street. First introduced by Hillier and Hanson [66,67] in the theory of space syntax and further developed by Turner et al. [68,69], there are two widely used metrics, i.e., angular closeness centrality (or *angular integration*, the potential of movement to a street segment) and angular betweenness centrality (or *angular choice*, the potential movement through a street segment). Several studies have used these metrics as proxies for walkability and reported associations between these metrics and health outcomes [16,17,18] and their ability to predict pedestrian movement [70]. As such, the method overcomes the oversimplification issues of alternative metrics that aim to capture the character of an urban area (e.g., ‘urbanicity’) by counting the number of intersections or measuring the aggregated population density.

The angular closeness centrality calculates the angular distance between every street segment and every other segment in the street network within a given radius, using the shortest angular path. The variable is calculated as follows (see [69] for a comprehensive description):

(5) $$C_{C}\left(p_{i}\right)={\left(\sum_{k}d_{ik}\right)}^{-1}\;,$$

where *d_jk_* is the length of the shortest path between node *p_i_* and *p_k_*. The angular betweenness centrality is calculated by generating the shortest paths between all segments within a given radius as follows:

(6) $$C_{B}\left(p_{i}\right)=\frac{\sum_{j}\sum_{k}g_{jk}\left(p_{i}\right)}{g_{jk}}\;\;\;\;\;\;\;\;\;(j<k),$$

where *g_jk_(p_i_)* is the number of shortest paths between node *p_j_* and *p_k_* which contain node *p_i_*, and *g_jk_* is the number of all the shortest paths between *p_j_* and *p_k_*. The base for this analysis was the network model using the OS Highways and OS Urban Path information described in Section 2.3.

We calculated the relative centrality (i.e., the angular closeness and angular betweenness centrality) for all the street segments in Bradford at varying radii. We measured the centralities at the residential segment and aggregated the average and maximum value within 300 m from a home address point.

#### 2.5.7. Built Form

A main concern of the field of urban planning and urban morphology is the development of methodologies to quantitatively capture differences in the urban form. We selected a prominent approach of these [71,72] and calculated a series of variables that described the spatial characteristic of urban densities and form, and combined these with descriptive information from secondary data (i.e., the Department for Levelling Up, and the Housing and Communities’ Energy Performance Certificate database). Specifically, we described built-form characteristics at the building level, as well as the block level. We measured the building footprint, building height, building volume, and building floor area utilising the OS MasterMap and Building Height data in combination with OS Highways information from 2021. This data will enable investigations between urban densities and health outcomes.

We calculated for each address the floor-space index (FSI), ground-space index (GSI), open-space ratio (OSR), and the average building layers of floors (L) for the block (i.e., the continuous area bounded by streets) that contained an address point (see [71] for a comprehensive description). We further included EPC-based information on the build form classification (i.e., detached, semi-detached, end-terrace, mid-terrace, enclosed end-terrace, and enclosed mid-terrace), the dwelling type (i.e., house, bungalow, flat, maisonette, and park home), the construction age band (i.e., before 1900, 1900–1929, 1930–1949, etc.), and the number of storeys and tenure type (i.e., rental (private), rental (social), and owner-occupied).

#### 2.5.8. Indoor Qualities

The COVID-19 pandemic has uncovered the importance of indoor qualities for physical and mental health [22,23], but capturing such indoor qualities at scale is a difficult task. For this reason, we selected a variety of variables of the EPC’s secondary information which could be used as a proxy for the indoor qualities. The variables included data on energy consumption, energy consumption potential, lighting, heating and hot water cost, size of glazed areas, floor area and height, number of heated rooms, number of habitable rooms, and the number of extensions.

#### 2.5.9. Food Environments

Numerous studies have reported associations between fast-food exposure and health outcomes such as obesity in adults and children; however, the evidence is mixed [37]. Such mixed evidence might be caused by differences and imprecisions in measuring the exposure, which often is based on crude area counts. To address such shortcomings, we measured the exposure to fast-food outlets at a high-spatial precision by calculating the accessibility as the walking distance from an address to fast-food outlets at varying distances, and then we calculated the proportion of fast-food to all food offerings around the home. For this, we adapted a method by [20] using 2021 secondary OS Points of Interest (POI) information to identify the fast-food outlets through the existing classification within the POI data in combination with text-based keywords applied to an outlet’s name (see Appendix B in [20] for the used keyword set).

We counted the number of all the food offerings, the fast-food offerings, the ratio between the fast-food outlets and all food offering outlets within varying radii, and calculated the distance decay weighted counts.

## 3. Results: Built Environment Indicators

The result of the aforementioned methodology is a set of more than 500 environmental indicators outlined in Table 1.

**Figure 4:**
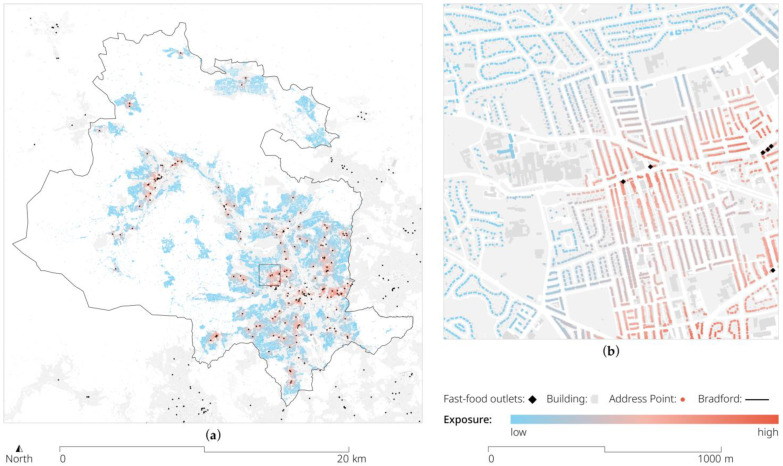
Visualisation of fast-food exposure measure showing distance decay weighted counts: (**a**) for the entire metropolitan district of Bradford; (**b**) detail zoom-in on Toller ward illustrating the address-level variation in the exposure measurement. © OS and Crown copyright 2022.

Figure 4 shows a visualisation of a variable from the food environment domain, i.e., the fast-food exposure using a distance decay weighted count. The mapping provides geographic insights into the spatial distribution of fast-food outlets and the difference in exposure for each resident in Bradford. Urbanised areas feature a disproportionate number of fast-food outlets with the highest level of exposure in neighbourhoods around the centre. Suburban and rural neighbourhoods, on the other hand, feature comparably less exposure.

**Figure 5:**
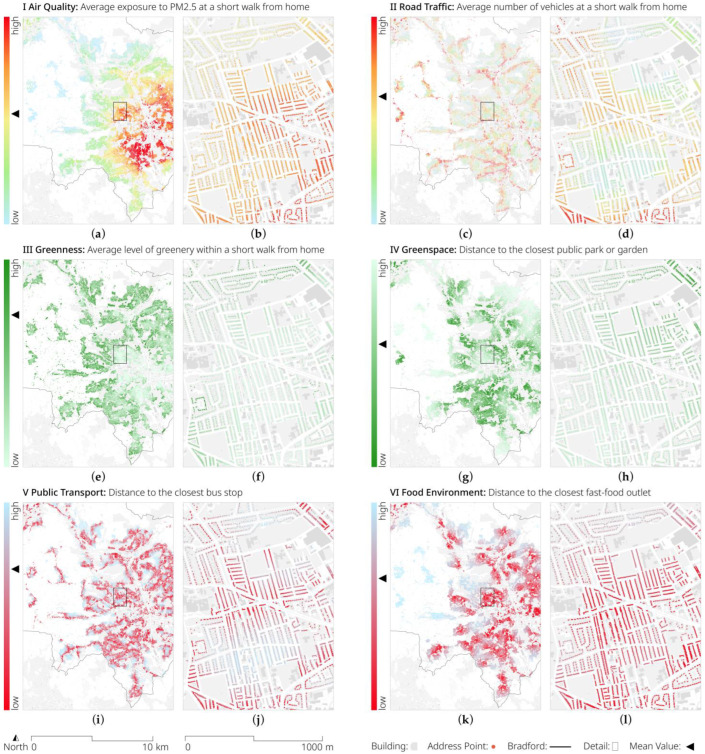
Visualisation of the selection of exemplary EIs (I–IV, see Table 2 for details) for the entire metropolitan district of Bradford and a detailed zoom-in on the Toller ward. EIs include: (**a**,**b**) average exposure to PM 2.5; (**c**,**d**) number of vehicles during the morning period; (**e**,**f**) average level of greenery; (**g**,**h**) distance to the closest public park or garden; (**i**,**j**) distance to the closest bus stop; and (**k**,**l**) distance to the closest fast-food outlet. © OS and Crown copyright 2022.

**Figure 6:**
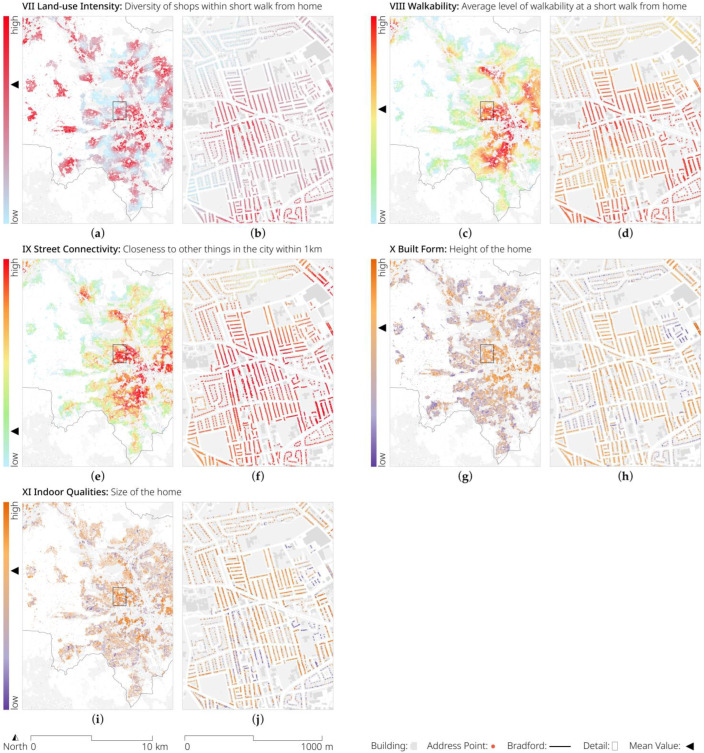
Visualisation of the selection of exemplary EIs (IX–XI, see Table 2 for details) for the entire metropolitan district of Bradford and a detailed zoom-in on the Toller ward. EIs include: (**a**,**b**) diversity of shops; (**c**,**d**) average level of walkability; (**e**,**f**) closeness to other things in the city; (**g**,**h**) height of the home; and (**i**,**j**) size of the home. © OS and Crown copyright 2022.

In addition to the insights into the geographic distribution of fast-food outlets, we selected 11 exemplary EIs (see Table 2) to illustrate the environmental exposure of an average person in Bradford. Figure 5 and Figure 6 show the mappings of these, demonstrating the potential insights that these data can provide both at city and local levels.

We combined this selection of exemplary indicators with demographic information from the 2021 Census to produce a synthetic person: Samina. Samina was a 12-year-old British Pakistani girl. She lived with her two parents in a 5.70 m high mid-terrace house of 78.00 square metres. During a short morning walk from home, she would be passed by 182 cars and exposed to 9.01 micrograms per cubic metre of particulate matter 2.5, which is comparable to the annual mean concentration in the UK at urban background monitoring sites in 2018 and just below the WHO guideline [73] of an annual mean of 10 micrograms per cubic metre. The streets that she would traverse would not be very walkable (0.84), feature little diversity of shops (0.06), and have a greenery level of 0.22, which is a relatively low green environment when compared to the highest values of places in Bradford. Samina would be close to public transport (182.99 m), but not very close to many other streets in the city (a 1419.43 street centrality), and closer to a fast-food outlet (561.89 m) than to a public park or garden (889.26 m).

While this is only a single example, it demonstrates how powerful such a dataset can be when multiplied across a cohort—or indeed a city’s population. Not only does it enable the researcher to detail each individual’s susceptibility to ecological influences on their health (and their confounders), but it also allows a policy-maker to identify—through geographical mapping of the data—where interventions, such as reducing the sources of pollution, or improving access to green spaces, should be targeted most urgently.

## 4. Discussion

Constructing large-scale, longitudinal, individual-level environment exposure variables poses a series of challenges, which we divide into a) general data challenges and b) measurement challenges. General data challenges include difficulties around the availability and comparability of historic data. Availability and comparability challenges can range from differences in quality, spatial resolution, precision, completion, and classification, while measurement challenges consist of fundamental questions touching upon definition, classification and operationalisation issues.

To address these, we selected datasets for which comparable alternative datasets are available globally and which are consistent throughout the years (see Table 3). Using data from the earliest available dataset in the years where otherwise no data is available constitutes a feasible alternative for datasets that show little temporal variation, such as the entrance points of parks (i.e., OS Open Greenspace). To provide an illustration of the comparability challenges, the OS AddressBase Premium data has been available since 2004; however, a consistent address classification has existed only since 2013. This means that while spatial information exists prior to 2013, the identification of residential or non-domestic functions will need to be inferred through the use of alternative classifications such as the Valuation Office Agency’s Primary Description and Special Category (Scat) Codes. This introduces comparability issues as the inferred classifications are not identical to the existing land-use classes.

Furthermore, we trialled variables from the food environment domain, i.e., exposure to fast-food in conjunction with data from the BiB longitudinal cohort survey. Specifically, we looked at the association between fast-food exposure and childhood obesity. For this, we first applied our outlined method and calculated the exposure variables for all the residential addresses and all the school addresses in Bradford, as well as journeys between the two. We then geo-referenced the BiB participants’ address information as well as the school address information and linked these to their respective UPRNs. These UPRNs were then used to link the environmental exposure information to the BiB participants in a safe data environment. Our analysis of the exposure around the home showed that increasing spatial precision in the quantification of the exposure to FFOs does not lead to differences in the associations with childhood obesity, which challenges the previous findings reporting associations between these two [74]. This analysis highlighted a series of measurement challenges. For example, there is little agreement as to what constitutes a fast-food outlet and differences in the definitions likely explain the heterogeneity in the reported associations [37]. In addition, a variety of potential spatial and non-spatial confounders which were not captured by our exposure measurements might be at play and would have to be controlled for when utilising environmental exposure variables. In the context of fast-food exposure, these could include, among others, a genetic predisposition, behavioural differences, financial situations, or level of deprivation. Each of these might also be expressed as a spatio-temporal urban self-selection process. For example, while obesity has been linked to economic deprivation, the density of fast-food outlets within reach of a home address seems not to be the main driving factor [75], which highlights the importance of controlling for such confounders when utilising spatial information, and it indeed points to the importance of measuring the proximity by street distance (rather than an average across an area, or ‘as the crow flies’).

The strength of our approach is—besides capturing the exposure at a spatial resolution at which it is perceived—its generalisability, which is applicable across the globe and to any type of environmental exposure through time. For example, future studies that wish to apply the proposed methodology in international contexts may consider the inclusion of additional variables relevant to the respective local environmental conditions, e.g., the surface temperature information derived from satellite imagery for areas affected by more extreme climates. The method enables not only investigations into the associations of exposure types and health outcomes but it provides the opportunity for causal inference research designs. An example of such a research design currently being undertaken by our researchers is to utilise involuntary house move information to investigate the causal effect of air pollution on respiratory diseases using the Connected Bradford database. Opportunities for causal inference research designs not only exist within the presented Connected Bradford database but for any longitudinal cohort survey. We have identified a series of longitudinal cohort surveys in the UK, i.e., the National Child Development Study (NCDS), 1970 British Cohort Study (BCS70), UK Household Longitudinal Study (UKHLS), British Household Panel Survey (BHPS), Millennium Cohort Study (MCS) and Next Step (previously known as the Longitudinal Study of Young People in England (LSYPE)) (see Table 4), which can adopt our approach by linking a participant’s address to their UPRNs (subject to the necessary approvals) and subsequently to their environmental exposure without compromising their identity. Such nationwide longitudinal cohort data provides an untapped potential in identifying the causal links between the built environment and health outcomes.

## 5. Conclusions

This paper outlined the theoretical and technical foundation of Connected Bradford’s environmental exposure indicators. The dataset constitutes a unique information source providing high-resolution geospatial information on the exposure to and within the built environment for the entire population of Bradford. Our street and building-level information have been captured at a scale that enables the formulation of guidelines and spatial planning policies for the modification of the built environment—a critical gap in the current knowledge. This effort will enable pivotal research into the relationship and causal links between the built environment and health, informing planning and policy-making. Moreover, it has the potential to serve as a template for nationwide replication. The recent *WHO priorities for urban health* report [1] emphasised the importance of building city-level evidence on urban environments and health outcomes in order to obtain “a clearer picture of the association between urban exposures and health across the life course”. We have proposed here a method for doing so that overcomes some fundamental challenges of capturing precise, meaningful data at a scale that can improve the quality of evidence for research in this urgent policy domain.

## Figures and Tables

**Table 1:** Overview of environmental indicators, exposure measurements and data sources.

No.	Environmental Domain	Exposure Measurement	Spatial Relationship	Distance Type and Radii	Data Source
I	Air Quality	Concentration of Particulate Matter (PM) 10, PM 2.5, and nitrogen oxides (NOx)	(**g**), (**i**)	(**1**), 100, 300, 500, 1000, 1500	Environmental modelling, City of Bradford, Defra
II	Road Traffic	Average traffic volume and level of congestion	(**b**), (**f**)	(**2**), 100, 300	Basemap Journey Time data
III	Greenness	Normalised Difference Vegetation Index (NDVI)	(**b**), (**i**)	(**1**), 100, 300, 500, 1000, 1500	NASA Landsat 8–9
IV	Greenspace	Accessibility of green spaces entrance points by class (e.g., play space, public park or garden, and religious grounds)	(**d**), (**e**), (**f**)	(**2**), (4), 300, 500, 1000, 2000, 5000	OS Open Greenspace
V	Public Transport	Accessibility of public transport stops by class (e.g., bus, metro, rail, and coach)	(**d**), (**e**), (**f**)	(**2**), (4), 300, 500, 1000, 2000, 5000	NaPTAN
VI	Food Environment	Accessibility of all food outlets, fast-food outlets, and ratio of fast-food outlets	(**d**), (**e**), (**f**), (**h**)	(**2**), (4), 300, 500, 1000, 2000, 5000	POI
VII	Land-use Intensity	Shannon’s Diversity Index (SDI)	(**g**), (**i**)	(**1**), 100, 300, 500, 1000, 1500	POI
VIII	Walkability	Walkability Index (WI)	(**g**), (**i**)	(**1**), 100, 300, 500, 1000, 1500	NaPTAN, POI, UK Census, OS Highways, and OS AddressBase
IX	Street Centrality	Betweenness and closeness centrality	(**b**), (**f**)	(**2**), 300, 500, 1000, 15,000, 2000	OS Highways
X	Built Form	Building footprint, building height, building volume, building floor area, floor space index (FSI), ground space index (GSI), open-space ratio (OSR), average building layers of floors (L), build form, construction age band, number of storeys, dwelling type, and tenure type	(**a**), (**c**)	-	OS MasterMap, OS Highways, and EPC
XI	Indoor Qualities	Energy consumption, lighting/heating/hot water cost, glazed area, floor area, number of heated rooms, number of habitable rooms, floor height, and number of extensions	(**a**)	-	EPC

**Table 2:** Selection of exemplary EIs from each of the 11 domains (Roman numerals match Table 1).

No.	Environmental Exposure Indicator *	Mean	Std. Dev.	Min.	Max.	Median	Mode
I	Avg. exposure to PM 2.5 at a short walk from home (μg/m^3^)	8.92	0.98	6.40	11.48	9.01	10.31
II	Avg. number of vehicles at a short walk from home (AM)	188.72	182.93	0.00	2268.00	141.00	0.00
III	Avg. level of greenery within a short walk from home	0.22	0.06	0.08	0.44	0.22	0.12
IV	Distance to the closest public park or garden (m)	1044.94	708.12	10.54	5715.32	889.26	1105.96
V	Distance to the closest bus stop (m)	216.52	155.05	10.05	4338.08	182.99	268.05
VI	Distance to the closest fast-food outlet (m)	763.99	739.79	10.32	5869.15	561.89	298.47
VII	Diversity of shops within a short walk from home	0.08	0.07	0.01	0.51	0.06	0.33
VIII	Avg. level of walkability within a short walk from home	0.86	0.37	0.05	2.51	0.84	2.03
IX	Closeness to other things in the city within 1 km	1578.83	931.49	0.36	5986.89	1419.43	4157.30
X	Height of the home building (m)	6.55	3.88	0.10	39.60	5.70	5.40
XI	Size of the home (sqm)	88.87	49.56	0.00	3384.00	78.00	70.00

* If not specified, the values are indices of the respective measurement.

**Table 3:** Overview of available datasets by year.

Dataset	2013	2014	2015	2016	2017	2018	2019	2020	2021	2022
OS MasterMap	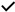	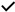	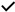	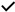	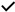	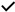	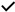	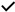	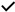	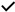
OS Highways (OS ITN)	( 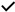 )	( 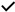 )	( 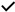 )	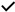	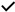	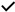	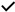	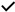	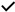	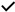
OS Urban Paths	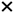	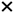	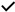	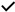	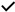	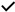	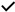	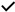	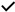	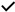
OS AddressBase Prem.	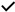	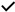	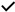	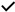	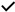	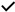	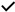	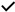	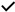	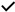
OS POI	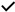	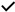	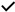	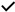	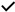	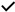	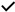	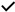	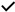	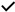
OS Open Greenspace	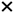	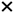	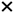	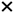	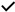	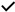	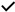	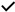	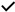	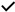
USGS Landsat 8	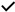	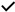	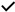	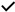	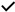	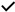	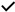	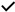	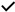	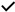
EPC	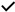	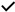	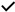	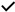	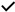	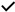	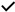	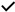	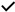	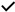
DEFRA (LA modelling)	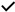	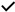	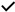	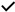	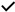	( 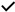 )	( 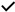 )	( 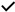 )	( 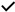 )	( 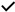 )
Trafficmaster	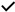	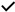	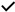	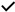	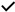	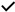	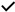	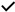	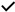	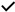
NaPTAN	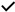	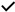	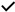	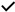	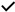	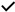	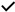	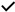	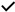	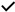

‘
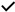
’: dataset is available, ‘(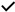)’: only dataset in brackets available, and ‘
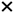
’: dataset is not available.

**Table 4:** Overview of sweeps of alternative British longitudinal cohort surveys.

Dataset	2013	2014	2015	2016	2017	2018	2019	2020	2021	2022
NCDS	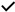	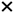	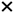	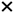	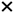	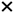	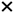	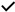	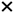	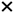
BCS70	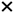	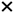	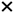	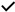	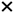	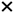	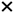	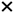	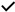	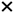
UKHLS	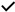	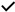	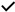	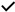	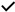	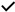	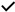	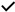	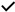	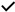
BHPS	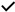	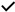	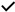	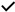	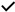	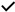	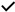	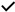	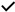	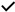
MCS	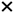	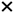	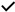	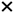	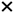	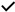	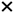	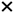	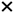	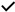
Next Steps (LSYPE)	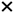	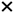	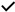	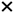	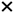	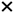	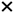	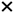	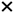	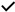

‘
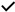
’: dataset is available, and ‘
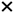
’: dataset is not available.

## Data Availability

The data described in this article is available on request due to restrictions regarding privacy and ethical concerns. Further details on how to apply for access to Connected Bradford data are available at https://www.bradfordresearch.nhs.uk/our-research-teams/connected-bradford/ (accessed on 14 December 2022).
